# Operationalizing respectful maternity care at the healthcare provider level: a systematic scoping review

**DOI:** 10.1186/s12978-021-01241-5

**Published:** 2021-10-01

**Authors:** R. Rima Jolivet, Jewel Gausman, Neena Kapoor, Ana Langer, Jigyasa Sharma, Katherine E. A. Semrau

**Affiliations:** 1 Department of Global Health and Population, Harvard TH Chan School of Public Health, 677 Huntington Avenue, Boston, MA 02115 USA; 2 BetterBirth Program, Ariadne Labs|Brigham and Women’s Hospital and Harvard TH Chan School of Public Health, Boston, MA USA; 3 Division of Global Health Equity, Brigham and Women’s Hospital, Boston, MA USA; 4 Department of Medicine, Harvard Medical School, 401 Park Drive, 3rd Floor West, Boston, MA 02215 USA

**Keywords:** Maternal health, Quality of care, Respectful maternity care, Professional guidelines, Obstetrics & gynecology, Nursing, Midwifery, Measurement

## Abstract

**Background:**

Ensuring the right to respectful care for maternal and newborn health, a critical dimension of quality and acceptability, requires meeting standards for Respectful Maternity Care (RMC). Absence of mistreatment does not constitute RMC. Evidence generation to inform definitional standards for RMC is in an early stage. The aim of this systematic review is clear provider-level operationalization of key RMC principles, to facilitate their consistent implementation.

**Methods:**

Two rights-based frameworks define the underlying principles of RMC. A qualitative synthesis of both frameworks resulted in seven fundamental rights during childbirth that form the foundation of RMC. To codify operational definitions for these key elements of RMC at the healthcare provider level, we systematically reviewed peer-reviewed literature, grey literature, white papers, and seminal documents on RMC. We focused on literature describing RMC in the affirmative rather than mistreatment experienced by women during childbirth, and operationalized RMC by describing objective provider-level behaviors.

**Results:**

Through a systematic review, 514 records (peer-reviewed articles, reports, and guidelines) were assessed to identify operational definitions of RMC grounded in those rights. After screening and review, 54 records were included in the qualitative synthesis and mapped to the seven RMC rights. The majority of articles provided guidance on operationalization of rights to freedom from harm and ill treatment; dignity and respect; information and informed consent; privacy and confidentiality; and timely healthcare. Only a quarter of articles mentioned concrete or affirmative actions to operationalize the right to non-discrimination, equality and equitable care; less than 15%, the right to liberty and freedom from coercion. Provider behaviors mentioned in the literature aligned overall with seven RMC principles; yet the smaller number of available research studies that included operationalized definitions for some key elements of RMC illustrates the nascent stage of evidence-generation in this area.

**Conclusions:**

Lack of systematic codification, grounded in empirical evidence, of operational definitions for RMC at the provider level has limited the study, design, implementation, and comparative assessment of respectful care. This qualitative systematic review provides a foundation for maternity healthcare professional policy, training, programming, research, and program evaluation aimed at studying and improving RMC at the provider level.

## Plain Language Summary

Respectful care for mothers and newborns is a right and important part of ensuring that their care is high quality and acceptable to them. Just because there is no mistreatment does not mean that Respectful Maternity Care (RMC) was given. Without a clear framework for provider behaviors that reflect RMC principles, it is hard to ensure every woman and newborn gets respectful care in practice. We compared and combined two frameworks summarizing maternal and newborn rights and came out with seven categories. Then we searched for articles that mentioned provider behaviors reflecting RMC. We found 514 articles and ended up with 54 after careful review, from which we pulled the observable behaviors for providers in each category. Almost all papers mentioned actions to protect women and newborns from harm and mistreatment, to treat them with dignity and respect, and to give information and respect choices. About half of papers mentioned actions to protect privacy and to make sure every mother and newborn gets care when needed. Only 25% of papers mentioned actions to make sure all women and newborns receive equal care, and only 15% included actions to make sure women and newborns are physically free to leave facilities at will, and get care whether or not they can pay. This framework defining RMC behaviors for providers is based on data from many studies and can be useful to look at whether maternal newborn care in facilities meets these standards and to inform training and more research to improve RMC.

## Background

Maternal mortality and morbidity are widely recognized as fundamental human rights issues, and women’s right to sexual and reproductive health care—including maternity care—that is available, accessible, acceptable, and of high quality (AAAQ) is a central tenet of the technical guidance issued by the Office of the United Nations High Commissioner for Human Rights (OHCHR) on a “Human rights-based approach to reduce preventable maternal morbidity and mortality.” [1] Moreover, the guidance states (p.3) that “Ensuring women’s sexual and reproductive health rights requires meeting standards with regard to health facilities, goods and services…” and stipulates that “respectful care for women using health services is a critical dimension of both quality and acceptability.” Yet, to date there is no consensus on evidence-based standards for Respectful Maternity Care (RMC).

A central focus of global maternal health efforts over the last decades has been to increase the number of women giving birth within health facilities, as a mechanism to increase skilled birth attendance [2]. As of 2019, approximately 76% of women globally delivered in a health facility [3]. However, the global push toward facility-based birth for all women in all countries has exposed health system deficiencies and brought to light the pervasive problem of mistreatment of women in the context of facility-based maternity care [4, 5]. Several qualitative and quantitative studies demonstrate a high prevalence of disrespect and mistreatment during childbirth, including verbal, physical, and sexual abuse [6–17]. Mistreatment of women and newborns during maternity care is not only a violation of their rights, but it can also be a deterrent to current and future skilled care utilization [11, 18]. Frontline maternity care providers are most often the perpetrators of such mistreatment; however, in many settings where the majority of care is provided by nurses and midwives, they themselves are also subject to disrespectful, untenable conditions and health system deficiencies that, in turn, drive disrespectful behavior and contribute to women’s poor experiences of care [19].

Bowser and Hill’s [11] landscape review describing and categorizing disrespectful and abusive care during childbirth was seminal in increasing visibility of this topic in policy and research settings. This work informed the development of the *Respectful Maternity Care Charter: Universal Rights of Mothers and Newborns* (RMC Charter) (2011, updated 2019) [20] and the World Health Organization statement on the prevention and elimination of disrespect and abuse during facility-based childbirth (2014) [21]. A subsequent systematic review and thematic analysis of the published literature on mistreatment in the context of facility-based by Bohren et al. [9], corroborated the Bowser & Hill typology and added attention to health system deficiencies. Ensuring RMC is now a key feature of the WHO vision for quality of care for mothers and newborns [5], and the WHO standards for improving quality of maternal and newborn care in health facilities [22]. Categories [11], prevalence [6, 8–10, 14, 17], and to some degree drivers [18, 19] of disrespect and abuse in the context of facility-based maternity care have been explored, and rights-based frameworks have been articulated [20, 23]. However, the absence of mistreatment in facility-based care does not in itself constitute RMC. While disrespect and abuse have been well defined and studied, the “positive dimension” of RMC has not been as well conceptualized, defined, described, or measured to date. Evidence generation to inform definitional standards for RMC is ongoing, and in an early stage of development. While examples of calls to action, programs, and approaches to RMC training are proliferating [24–26], to our knowledge no synthesis of provider-level standards for RMC has been put forward.

Frontline maternity care providers, through their intimate interactions with women and newborns during labor and delivery, are uniquely positioned to influence women’s experience of care, both as potential perpetrators of disrespect and abuse or change-agents for instituting RMC [11, 21]. Bowser and Hill [11] identify four provider-level mechanisms that can contribute to disrespect and abuse in facility-based childbirth: (1) provider prejudice and discriminatory behavior against certain sub-groups of women; (2) provider distancing from clients because of training that encourages social distance and normalizes disrespectful or abusive care; (3) provider demoralization because of weak health systems, human resource shortages, limited professional development opportunities; and (4) an atmosphere of disrespect and abuse between providers translating into abuse and disrespect of patients. Intervening at the provider level to support positive changes in provider behavior, within the context of healthy clinical environments and strengthened health systems, is therefore essential to ensure that all women have access to respectful care from competent providers. Consensus on evidence-informed provider-level operational definitions for RMC would provide a basis for such interventions.

We conducted this systematic evidence synthesis as the first part of a larger project to explore whether essential elements of RMC are included in professional practice standards for frontline maternity care professionals (forthcoming publication). In this first step, to develop an operational definition at of essential elements of RMC at maternity care provider-level, we reviewed the literature in two distinct, but related, phases.

## Methods

### Frameworks defining rights of women during childbirth

We began by reviewing seminal literature codifying, setting standards and guidelines, and identifying the rights of women to receive respectful care during childbirth. We focused on rights-based frameworks for two reasons: first and foremost, to highlight the essential rights-based dimension of RMC as per OHCHR technical guidance and secondly, because of a dearth of clinical or professional behavioral frameworks for RMC grounded in evidence. We identified seven seminal, definitional frameworks [9, 11, 20–23, 27] that outline a broad understanding of mistreatment of women (also referred to as disrespect and abuse) during facility-based childbirth and refer to RMC in the affirmative. Given that the objective of this systematic review was to identify categories of RMC and their operational definitions, we focused on literature that described RMC in the affirmative rather than describing the categories of mistreatment experienced by women during childbirth. On this basis, we narrowed the results of our review to two seminal works that both codify the rights to RMC during childbirth [20, 23].

The first framework, the RMC Charter, developed by the White Ribbon Alliance based on widely recognized global and regional human rights instruments, situates maternal and newborn health rights within the broader context of human rights [20]. The original charter identified seven rights of childbearing women, each corresponding to one of the categories of disrespect and abuse identified in the landscape review by Bowser and Hill. Of note, there was an update to the RMC Charter in 2019 that retained the original seven rights and added three more: the right of newborns to stay with their parent or guardian, the right to have their national identity recognized from birth, and the right to adequate nutrition, and water, sanitation and hygiene (WASH) in facilities. For this analysis, we utilized the original RMC Charter framework because two out of three of the newly added rights must be operationalized at the health system or policy level rather than the provider-level; and the third, the non-separation of the mother-baby pair, is addressed in the original RMC Charter.

The second framework from Khosla and colleagues [23] similarly mapped international human rights standards from a scoping review of human rights instruments to the corresponding categories of mistreatment of women during childbirth in facility settings that were identified in the later systematic review of mistreatment of women during facility-based childbirth by Bohren et al. [9].

Two reviewers (JS and RRJ) performed a head-to-head comparison of the rights identified in the two frameworks to compile a list of unique categories of the rights of women during pregnancy and childbirth. Using the synthesized categories of RMC from these two frameworks, we initiated a systematic review to operationalize the RMC categories through the description and cataloguing of actionable elements and observable behaviors for each category.

### Operational definition of respectful maternity care

Four reviewers (RRJ, JG, NK, and KEAS) then systematically reviewed peer-reviewed literature, grey literature, white papers and seminal documents on setting RMC standards to identify an operational definition of RMC at provider-level and its key elements within those previously established seven rights-based categories of RMC.

Following the PRISMA methodology [28], we searched electronic databases of peer-reviewed articles (Medline [via PubMed]). We conducted a Google Scholar search for grey literature and white papers. We also searched the Columbia University Mailman School of Public Health Averting Maternal Death and Disability (AMDD) program’s monthly RMC literature and media summaries from 2017 to 2020, which capture published reports from non-governmental organizations, international organizations, or ministries of health, as well as the World Health Organization website for content related to RMC. Additionally, we hand-searched bibliographies of relevant articles to ensure that key documents with RMC content are represented. All articles identified from different sources were imported into EndNote.

Our search string (limited to humans) was: *(((((mistreatment[All Fields] AND ("women"[MeSH Terms] OR "women"[All Fields]) AND ("parturition"[MeSH Terms] OR "parturition"[All Fields] OR "childbirth"[All Fields])) OR "disrespect and abuse"[All Fields]) OR (dehumanized[All Fields] AND care[All Fields])) OR (humanized[All Fields] AND care[All Fields])) OR "obstetric violence"[All Fields]) OR "respectful maternity care"[All Fields] AND ((("pregnancy"[MeSH Terms] OR "pregnancy"[All Fields]) OR ("parturition"[MeSH Terms] OR "parturition"[All Fields] OR "childbirth"[All Fields])) OR maternity[All Fields]).*

A PubMed search that was conducted using this string, with no start date and an end date of May 31, 2020, yielded 466 unique records. An additional 48 relevant articles were identified from supplemental hand searches as described. Forty-seven duplicates were removed. Thus, 467 articles were screened.

Two reviewers (NK and RRJ) screened titles and abstracts of all citations and two reviewers (JG and NK) reviewed the grey literature retrieved through hand search and the AMDD summaries. Articles were excluded if: (1) they lacked operationalized descriptions of RMC-related behaviors at the provider level; (2) they described the categories of mistreatment experienced by women during childbirth rather than respectful care behaviors in the affirmative; (3) they were not published in English or did not include an English translation; (4) they did not address facility-based childbirth. No exclusions were made on the basis of study design or study quality. The number of records excluded (along with the reason for exclusion) was documented. The full text of potentially eligible articles were independently reviewed by two reviewers (NK, RRJ or KEAS). Any discordance between two reviewers during both title and abstract screening and full-text review was resolved through discussion among all three reviewers.

Data for qualitative synthesis were extracted from the final list of articles by two independent reviewers (RRJ and KEAS) using a standardized form developed based on the categories of RMC previously defined. From each article, the examples and descriptions of behaviors that providers can/should adopt to exemplify respectful care were identified and extracted. These data were combined, discussed, and synthesized to operationalize each category of RMC.

## Results

### Frameworks defining rights of women during childbirth

The head-to-head comparison of the two frameworks utilized to summarize the rights of women during childbirth [20, 23] displayed significant overlap, with variation in the level of detail provided (Table 1). When the two frameworks were compared, seven key categories of RMC during childbirth emerged. The seven key categories focused on (1) right to be free from harm and ill treatment; (2) right to dignity and respect; (3) right to information, informed consent, respect for choices and preferences, including the right to companionship of choice where ever possible; (4) right to privacy and confidentiality; (5) right to non-discrimination, equality and equitable care; (6) right to timely healthcare and to the highest attainable level of health; and (7) right to liberty, autonomy, self-determination and freedom from coercion. Two domains identified in the framework by Khosla et al. were omitted from our analysis because they were not a provider-level obligation (right to an effective remedy) or not directly applicable during childbirth (right to decide the number, spacing, and timing of children).

**Table 1:** Head-to-head (direct) comparison of two frameworks defining rights of women during childbirth

Categories of Respectful Care during Childbirth Identified	White ribbon alliance [20]: Respectful Maternity Care: The Universal Rights of Childbearing Women (White Ribbon Alliance)	Khosla et al. [23]: International Human Rights and the Mistreatment of Women during Childbirth (World Health Organization)
RMC I. Right to be free from harm (violence, torture, harmful practices) and ill treatment (physical, sexual and verbal abuse)	Freedom from harm and ill treatment	Right to be free from violence
		Right to be free from torture and other ill-treatment
		Right to be free from practices that harm women and girls
RMC II. Right to dignity and respect	Dignity, respect	
RMC III. Right to information, informed consent and refusal, and respect for choices and preferences, including the right to companionship of choice wherever possible	Right to information, informed consent and refusal, and respect for choices and preferences, including the right to companionship of choice wherever possible	Right to information
RMC IV. Right to privacy and confidentiality	Confidentiality, privacy	Right to privacy
RMC V. Right to non-discrimination, equality and equitable care	Equality, freedom from discrimination, equitable care	Right to non-discrimination
RMC VI. Right to timely healthcare and to the highest attainable level of health	Right to timely healthcare and to the highest attainable level of health	Right to health
RMC VII. Right to liberty, autonomy, self-determination, and freedom from coercion	Liberty, autonomy, self-determination, and freedom from coercion	
*Excluded: not a provider-level obligation*		Right to an effective remedy
*Excluded: not during childbirth*		Right to decide the number, spacing and timing of children

### Operational definition of respectful maternity care

**Fig. 1:**
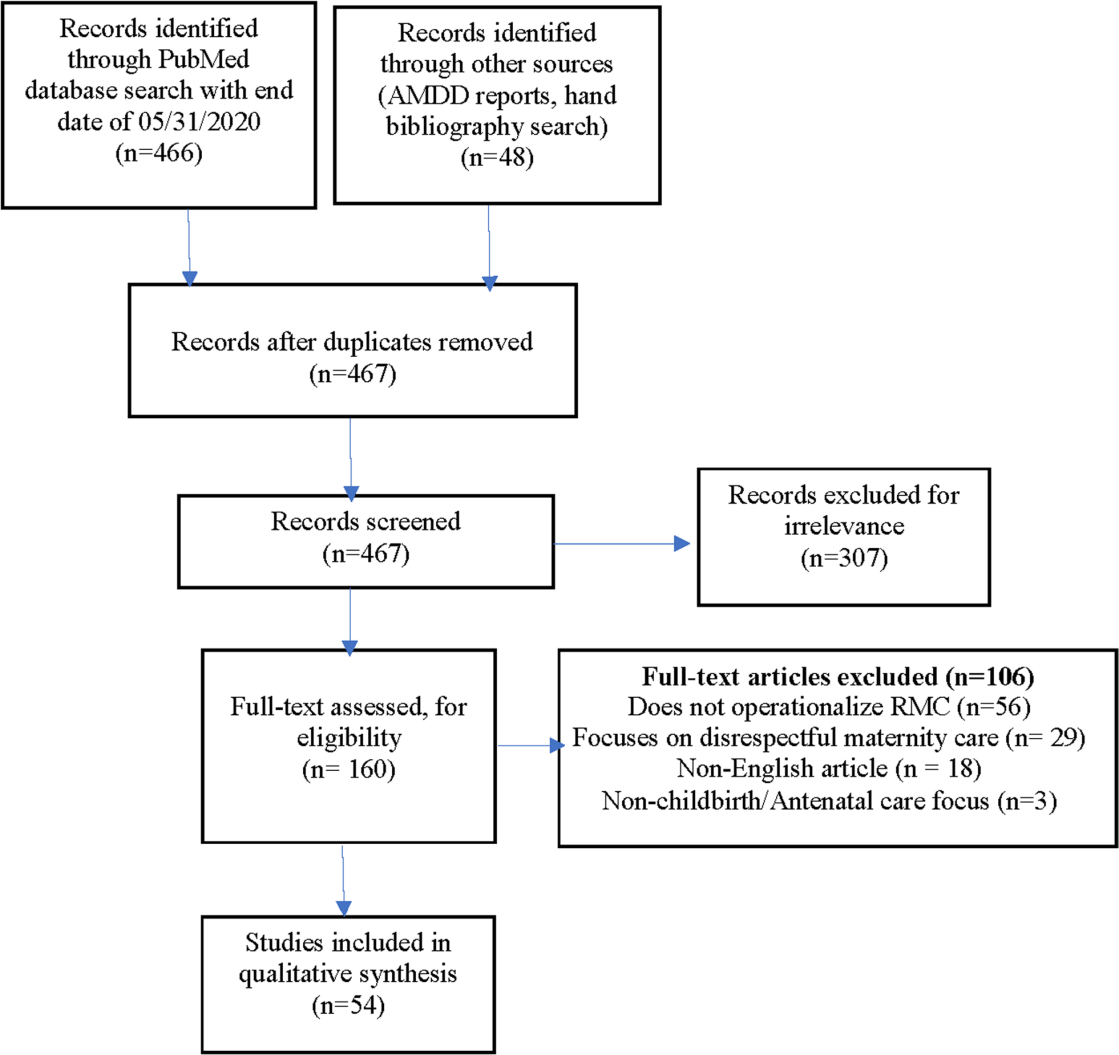
Flow diagram showing the study selection process to identify an operational definition of respectful maternity care for frontline healthcare providers, *1980-May 2020*

A total of 466 peer-reviewed articles were retrieved through electronic database search (Medline [via PubMed]) conducted on August 19, 2020. An additional 48 records were identified through Google Scholar, the World Health Organization website, the AMDD monthly RMC summaries from 2017 to 2020, and hand searching of bibliographies of relevant articles. After removing duplicates, we screened titles and abstracts of 467 records. At this stage, 307 records were excluded as irrelevant because they did not have an explicit mention of RMC related content. We reviewed full-text of 160 records, of which 106 were excluded for the following reasons: they did not operationalize RMC (n = 56); they focused solely on disrespect and abuse (n = 29); they were not in English (n = 18); or they focused on pregnancy care only and did not include facility-based childbirth (n = 3). The remaining 54 studies were selected for data extraction and qualitative evidence synthesis (Fig. 1).

From the themes identified across the two key frameworks, we documented the operationalized elements of RMC during childbirth from the 54 articles to each of the seven themes (Table 2). Most operational definitions focused on the relationship and care provided by clinicians to the woman and her newborn(s). However, in most environments, maternity care involves a team; thus, some themes extended the operational definitions of RMC to encompass interactions between providers. Here, we present each theme and their associated operationalized approaches.

**Table 2:** Operationalization of categories of respectful care during childbirth, for frontline healthcare workers providing maternity care

Categories of respectful care during childbirth	Operationalization of the categories of respectful care during childbirth	References
Right to be free from harm (violence, torture, harmful practices) and ill treatment (physical, sexual and verbal abuse)	1. Doctors/nurses/midwives provide only medically-indicated, evidence-based interventions 2. Doctors/nurses/midwives avoid harmful practices, including: a. Overuse of interventions, drugs and technology, b. Unnecessary separation of mother and baby 3. Doctors/nurses/midwives protect their clients from individual and institutional violence so that no client is subjected to abuse or mistreatment, including: a. physical, b. sexual, or c. verbal 4. Doctors/nurses/midwives provide: a. Food and fluids to women in normal labor b. Encouragement for early breastfeeding, including skin-to-skin contact with baby, immediately postpartum 5. Doctors/nurses/midwives provide to women in labor and birth (to strengthen their capabilities): a. Pharmacological and b. Non-pharmacological pain relief options and supportive care	Reis et al. [36]; International Federation of Gynecology and Obstetrics and others. [27]; Rosen et al. [37]; Thompson et al. [38]; Warren [15]; Miller et al. [39]; Sheferaw et al. [40]; Sheferaw [41] ; World Health Organization [22]; Kujawski [42]; Oosthuizen [43]; Asefa [44]; Bohren [45]; Dynes [46]; Shakibazadeh [47]; Taavoni [48]; Wassihun [49]; World Health Organization [50]; Afulani [51, 52]; Giordano [53]; Oliveira [54]; Perkins [55]; Afulani [56]; Bante [57]; Bohren [58]; Butler [59]; Lothian [24]; Montoya [60]; Moridi [61]; Devries [62] Page [63] Wagner [64] GarciadeLimaParada [65] Behruzi [66] Behruzi [67] Behruzi [68] Binfa [69] Ouedraogo [70] ConesaFerrer [71] Binfa [72]
Right to dignity and respect	1. Doctors/nurses/midwives provide culturally competent care, including respect for beliefs, traditions and culture 2. Doctors/nurses/midwives treat every client with respect, including respect for their personhood, experiences, and feelings 3. Doctors/nurses/midwives treat each other and all other cadres of collaborating providers and staff with respect 4. Doctors/nurses/midwives communicate effectively with clients, by: a. Using language that is respectful and positive, b. Using language they can understand, c. Greeting and addressing women politely and by name, and d. Providing verbal support and encouragement 5. Doctors/nurses/midwives provide positive, supportive non-verbal communication to clients 6. Doctors/nurses/midwives demonstrate sensitivity and empathy for women and partners experiencing loss and bereavement	Reis et al. [36]; Warren et al. [73]; International Federation of Gynecology and Obstetrics and others. [27]; Rosen et al. [37]; Thompson et al. [38]; Warren [15]; Miller et al. [39]; Patel et al. [74]; Sheferaw et al. [40]; Solnes et al. [75]; World Health Organization [22]; Kambala [76]; Kujawski [42]; Ndwiga [77]; Oosthuizen [43]; Sheferaw [41]; Vedam et al. [78], Vedam et al. [79]; Asefa [44]; Bohren [45]; Dynes [46]; Shakibazadeh [47]; Taavoni [48]; Wassihun [49]; World Health Organization [50]; Afulani [51, 52, 80]; Feijen-deJong [81]; Giordano [53]; Oliveira [54]; Afulani [56]; Ayoubi [82]; Bante [57]; Bohren [58]; Butler [59]; Lothian [24]; Moridi [61]; Montoya [60]; Page [63] Jorge [83] Guimaraes [84] Behruzi [66] [68] Behruzi 2011 Binfa [69] Ouedraogo [70] ConesaFerrer [71] Binfa [72] Lokugamage [85]
Right to information, informed consent and refusal, and respect for choices and preferences, including the right to companionship of choice wherever possible	1. Doctors/nurses/midwives encourage and support women to: a. Move freely during labor and b. Assume the position of their choice for birth 2. Doctors/nurses/midwives offer women the option to experience labor and birth with the companion of their choice and involve their family members in care and decisions if desired 3. Doctors/nurses/midwives provide information to clients about their health and care options, including: a. What to expect during labor and birth,, postpartum and newborn care b. Information on proposed interventions, tests and treatments, and c. Any out-of-pocket costs of care to be provided 4. Doctors/nurses/midwives encourage clients to: a. Ask questions and b. Express opinions or concerns 5. Doctors/nurses/midwives: a. Provide honest and complete information, b .Involve clients in decision making about their care, c. Solicit consent for all interventions, and d. Respect choices including refusal of interventions	Reis et al. [36]; Warren et al. [73]; International Federation of Gynecology and Obstetrics and others. [27]; Rosen et al. [37]; Thompson et al. [38]; Warren [15]; Miller et al. [39]; Patel et al. [74]; Sheferaw et al. [40]; Solnes et al. [75]; World Health Organization [22]; Kambala [76]; Kujawski [42]; Ndwiga [77]; Oosthuizen [43]; Sheferaw [41]; Vedam et al. [78, 79]; Asefa [44]; Bohren [45]; Dynes [46]; Shakibazadeh [47]; Taavoni [48]; World Health Organization [50]; Afulani [51, 52, 80]; Feijen-deJong [81]; Giordano [53]; Perkins [55]; Afulani [56]; Ayoubi [82]; Bohren [58]; Butler [59]; Lothian [24]; Moridi [61]; Montoya [60]; Page [63] Wagner [64] Guimaraes [84] GarciadeLimaParada [65] Behruzi [66, 67] Behruzi [68] Binfa [69] Ouedraogo [70] ConesaFerrer [71] Binfa [72] Lokugamage [85]
Right to privacy and confidentiality	1. Doctors/nurses/midwives provide visual and auditory privacy to clients during labor and birth, e.g., by providing care in a private room, or using curtains, screens, or drapes, and limiting the people present to those clinically indicated or desired by the woman 2. Doctors/nurses/midwives keep patient information confidential and do not share patient information unless indicated for the provision of effective care	Warren et al. [73]; International Federation of Gynecology and Obstetrics and others. [27]; Rosen et al. [37]; Thompson et al. [38]; Miller et al. [39]; Patel et al. [74]; Solnes et al. [75]; World Health Organization [22]; Kambala [76]; Kujawski [42]; Ndwiga [77]; Vedam et al. [78, 79]; Asefa [44]; Bohren [45]; Dynes [46]; Shakibazadeh [47]; Taavoni [48]; World Health Organization [50]; Afulani [51, 52, 80]; Giordano [53]; Afulani [56]; [82] Ayoubi 2020; [24, 59–61] Butler; Lothian; Moridi; Montoya; Behruzi [67] Ouedraogo [70] ConesaFerrer [71]
Right to non-discrimination, equality and equitable care	1. Doctors/nurses/midwives adhere to policies on non-discrimination 2. Doctors/nurses/midwives treat every client with equal respect and dignity, regardless of any specific personal attributes, including but not limited: to age, wealth, class, education, race or ethnicity, religion, LGBTQ+ , HIV or other health status	Warren et al. [15, 73] International Federation of Gynecology and Obstetrics and others. [27]; Solnes et al. [75]; World Health Organization [22]; Vedam et al. [79] Shakibazadeh [47]; Afulani; Ayoubi; Bohren; Butler; Lothian [24, 56, 58, 59, 82] Moridi [61]
Right to timely healthcare and to the highest attainable level of health	1. Doctors/nurses/midwives provide prompt attention and are responsive to clients’ needs for: a. Medical care and b. Comfort care 2. Doctors/nurses/midwives ensure that every woman has a skilled attendant present at her birth 3. Doctors/nurses/midwives ensure that no client is neglected or denied needed care, regardless of ability to pay 4. Doctors/nurses/midwives ensure continuity of care by coordinating effectively across settings and between providers	Reis et al. [36]; Warren et al. [15, 73] International Federation of Gynecology and Obstetrics and others. [27]; Sheferaw et al. [40]; Solnes et al. [75]; Kambala; Kujawski; Ndwiga, Oosthuizen [42, 43, 76, 77]; Asefa; Bohren; Dynes; Shakibazadeh; Taavoni; Wassihun; World Health Organization [44–50]; Afulani; Afulani; Ayoubi; Bante; Butler; Lothian; Moridi; [24, 52, 56, 57, 59, 61, 80, 82] Behruzi [68] Ouedraogo [70] Binfa [72]
Right to liberty, autonomy, self-determination, and freedom from coercion	1. Doctors/nurses/midwives do not illegally detain or physically restrain clients in the facility for any reason, including inability to pay 2. Doctors/nurses/midwives do not prevent clients from seeing or holding their babies for any reason, including inability to pay	Reis et al. [36]; International Federation of Gynecology and Obstetrics and others. [27]; World Health Organization [22]; Ndwiga [77] Taavoni [48]; Afulani [56]; Lothian [24];

*RMC I: Right to be free from harm and ill treatment*. Forty-one of the 54 articles provided guidance on how providers can ensure a woman’s right to be free from harm, including violence, torture, harmful practices and ill treatment (physical, sexual and verbal abuse). The five behaviors that providers can perform under this theme focused on provision of appropriate care and avoidance of inappropriate practices. Providers should: (1) provide only medically-indicated, evidence-based interventions; (2) avoid harmful practices including, overuse of interventions, drugs, and technology, and unnecessary separation of the mother and baby; (3) protect clients from individual and institutional violence and mistreatment, including physical, sexual, and verbal abuse. Clinicians should provide (4) food and fluids to women in normal labor and encouragement for early breastfeeding, including skin-to-skin contact with baby, immediately postpartum, as well as (5) pharmacological and non-pharmacological pain relief options and supportive care.

*RMC II: Right to dignity & respect.* Forty-nine out of 54 articles described behaviors to uphold the right to dignity and respect within the context of facility-based childbirth, including the importance of respect within inter-provider relationships. Important areas of RMC operationalization in this category focused on: (1) provision of culturally competent care, including respect for beliefs, traditions and culture; (2) respectful treatment of all clients, including respect for clients’ personhood, experiences, and feelings; and (3) respectful treatment of other clinicians and all other cadres of collaborating providers and staff. Further, providers should communicate effectively (4) by using language that clients can understand, and that is respectful and polite; greeting and addressing clients politely and by name; and providing verbal support and encouragement. Positive, supportive non-verbal communication to clients (5) is another important behavior exemplifying the right to dignity and respect. Finally, respect and dignity are demonstrated through sensitivity and empathy for women and partners experiencing loss and bereavement (6).

*RMC III: Right to information, informed consent and refusal, and respect for choices and preferences.* Forty-nine out of 54 in the systematic review highlighted the importance of ensuring women are provided information and the opportunity to give informed consent or refusal, and have their choices/decisions respected. Reflecting this right, operationally, (1) providers can encourage and support women to move freely during labor and birth and assume the position of their choice; and, (2) present women with the option to experience labor and birth with the companion of their choice and to involve their significant others in their care and decisions if they desire. Further, respecting the right to information extends beyond clinical or health information to encompass information about the cost of care. Clinicians should (3) provide information to women about their care options, what to expect during labor, birth and the postpartum period; information on proposed interventions, tests, and treatments; and any out-of-pocket costs. As part of enabling of the right to information and choice, clinicians should (4) provide honest and complete information, encourage women to ask questions and express their concerns and opinions, as well as (5) engage women with decision making about their care, solicit consent for all interventions, and respect their choices including refusal of interventions.

*RMC IV: Right to privacy and confidentiality*. A narrow majority of articles (32 out of 54) included reference to the importance of the right to (1) privacy and (2) confidentiality. Providers should keep patient information confidential and not share information unless indicated for the provision of effective care. In the synthesis, privacy and confidentiality were operationalized beyond sharing details of a medical record. Operationalizing the right to privacy and confidentiality focused on providing visual and auditory privacy to clients, including the use of drapes, screens, private room, etc., as well as limiting the number of people present to those clinically indicated or desired by the woman.

*RMC V: Right to non-discrimination, equality and equitable care*. Far fewer articles, thirteen out of 54 records, identified the importance of non-discrimination, equality and equitable care that were focused on (1) adhering to policies on non-discrimination. Further, providers should (2) treat every client with equal respect and dignity, regardless of specific personal attributes including, but not limited to age, wealth, class, education, race or ethnicity, religion, LGBTQI + , and health or HIV status.

*RMC VI: Right to timely healthcare and to the highest attainable level of health*. Twenty-eight records out of 54 noted the right to timely healthcare that focused on providers (1) giving prompt attention and being responsive to clients’ needs for medical care and comfort care. A critical component to operationalizing this right is (2) ensuring that every woman has a skilled birth attendant present at birth. Additionally, providers should (3) guarantee that no client is neglected or denied necessary care based on ability of pay. Finally, providers should (4) ensure continuity of care by coordinating across facilities/sites or settings and between providers.

*RMC VII: Right to liberty, autonomy, self-determination and freedom from coercion.* Only seven articles out of 54 focused on the right to liberty and self-determination. Two key operationalized actions emerged. Providers should not illegally detain or physically restrain women or their families in the facility for any reason, including inability to pay. Second, women should never be prevented from holding, seeing, or being with their newborn for any reason, including inability to pay.

## Discussion

RMC is a human right and a widely recognized core component of quality care [5, 20, 23]. Although articulation of the right to RMC is aligned around seven key rights principles, the operationalization of each principle within the context of healthcare professional behavior has been limited and disjointed. Grounded in two seminal rights-based documents defining the critical categories of RMC and using a systematic review of peer-reviewed and grey literature, we propose actionable practices and behaviors to operationalize RMC for maternity care providers. Global standard setting, professional guideline development, and program implementation can be clarified with this consolidated, evidence-informed set of key functions to enable and empower RMC by providers and clinicians.

In the systematic review, 54 articles were identified that described objective behaviors or concrete guidance and steps to meet the seven key principles of RMC. However, most of the articles provided insights on behaviors aimed at enacting three out of the seven key principles: (1) the right to be free from harm and ill treatment; (2) the right to dignity and respect; (3) the right to information, informed consent and refusal, and respect for choices and preferences. Fewer than two-thirds of articles referenced behaviors reflecting the right to privacy and confidentiality, although lack of privacy has been found to be a barrier to facility care across numerous studies [18]. Furthermore, only roughly half of articles reflected actions to uphold the right to timely healthcare and to the highest attainable level of health. Given the attention to increasing skilled birth attendance globally [29], it is surprising that more articles focused on RMC did not emphasize the right to timely care and attendance during birth. Moreover, less than a quarter of the articles reviewed specified provider behaviors aimed at ensuring the right to non-discrimination, equality and equitable care. Given the salience of health disparities in maternity care in terms of coverage of key interventions, quality of care, experiences of care, and outcomes of care, both within and across countries, this finding has important implications and emphasizes the need for more attention to operationalizing RMC in this area [30–33]. In addition, less than 15% of articles reviewed reflected the right to liberty, bodily autonomy, self-determination and freedom from coercion. The egregiousness of the harm caused to women, infants, and families from detention in childbirth facilities and evidence that suggests such behavior can be driven by individual, ad hoc judgments at provider and staff level in weak facilities [34] warrants provider-level accountability as duty-bearers to uphold and fulfill this right. The lack of available literature that included operationalized definitions corresponding to these two categories of RMC illustrates the nascent stage of evidence in this area.

There are some key strengths and limitations to this analysis. To our knowledge, this is the first systematic review of provider behaviors constituting RMC; furthermore, it rests on a firm conceptual foundation provided by two highly convergent definitional frameworks enumerating key RMC principles [20, 23]. An additional strength is the consistency of the operational definitions identified. Over the course of the development of the review, numerous new articles were released that discuss RMC, reflecting growing interest and ongoing efforts in this area; nevertheless, the foundational principles of RMC have not been altered. Not surprisingly, given the greater emphasis on health facility deficiencies in the influential 2015 systematic review by Bohren et al. [9], a notable addition to the seven essential elements of RMC in more recent literature is the evaluation of health system and facility characteristics. The addition of this new dimension did not change our operational definitions of RMC for the purpose of this review, given our focus on provider-level behaviors. One potential limitation, as with any systematic review, is that some relevant literature may not have been captured if our search terms were not comprehensive, and because of the exclusion of non-English articles. To address this limitation at least partly our search strategy included a systematic search of databases, a bibliographic search, and review of the grey literature. Of the 106 full-text articles reviewed and excluded, only 18 were excluded because no English translation was available.

### Implications for practice

In proposing a codification of actionable and operational definitions for the fundamental principles of RMC based on evidence, this qualitative systematic review provides a foundation for maternity healthcare professional policy, training, programming, and program evaluation aimed at studying and improving RMC at the provider level. Across diverse settings, context-specific interpretations and expressions of these provider-level behaviors may be needed to fully operationalize RMC II: the right to dignity and respect, particularly, in its aspect related to the provision of culturally competent care, including respect for beliefs, traditions and culture.

### Implications for research

For the research community, these operational functions and definitions of RMC can provide a launching point for validation as well as a common lexicon and basis for measurement and assessment of RMC. Currently, assessment of RMC has been approached using varied definitions and methods, including observation of childbirth and post-childbirth interviews with women. Potentially, this list of functional RMC actions broadens the scope for assessment and provides practical care steps to be monitored. Further, indicators built around these operationalized definitions can contribute to the assessment of effective coverage of high quality childbirth care [35].

## Conclusions

It is hoped that this review and synthesis will contribute toward an evidence-based foundation for provider level interventions to improve the delivery of respectful maternity care. The systematic codification, grounded in evidence, of operational definitions for RMC at the provider level should facilitate the study, design, implementation, and comparative assessment of respectful care.

## Data Availability

The data supporting the conclusions of this article are included within the article.

## Abbreviations

AAAQ: Available, accessible, acceptable, quality
AMDD: Averting Maternal Death and Disability
RMC: Respectful Maternity Care
